# Clinical factors that influence the occurrence of symptomatic pseudoaneurysms and arteriovenous fistulas after partial nephrectomy: multi-institutional study of renal function outcomes after one year of selective arterial embolization

**DOI:** 10.1590/S1677-5538.IBJU.2019.0789

**Published:** 2020-11-18

**Authors:** Chan Ho Lee, Hong Koo Ha, Ja Yoon Ku, Won Ik Seo, Seock Hwan Choi

**Affiliations:** 1 Inje University College of Medicine Inje University Busan Paik Hospital Department of Urology Busan Republic of Korea Department of Urology, Inje University Busan Paik Hospital, Inje University College of Medicine, Busan, Republic of Korea; 2 Pusan National University School of Medicine Pusan National University Hospital Department of Urology Busan Republic of Korea Department of Urology, Pusan National University Hospital, Pusan National University School of Medicine, Busan, Republic of Korea; 3 Kyungpook National University School of Medicine Department of Urology Daegu Republic of Korea Department of Urology, School of Medicine, Kyungpook National University, Daegu, Republic of Korea

**Keywords:** Aneurysm, False, Nephrectomy, Arteriovenous Fistula

## Abstract

**Purpose:**

Renal artery pseudoaneurysms (RAPs) and arteriovenous fistulas (AVFs) are rare but potentially life-threatening complications after partial nephrectomy (PN). Selective arterial embolization (SAE) is an effective method for controlling RAPs/AVFs. We assessed the clinical factors affecting the occurrence of RAPs/AVFs after PN and the effects of SAE on postsurgical renal function.

**Materials and Methods:**

Four hundred ninety-three patients who underwent PN were retrospectively reviewed. They were placed in either the SAE or the non-SAE group. The effects of clinical factors, including R.E.N.A.L. scores, on the occurrence of RAPs/AVFs were analyzed. The influence of SAE on the estimated glomerular filtration rate (eGFR) during the first postoperative year was evaluated.

**Results:**

Thirty-three (6.7%) patients experienced RAPs/AVFs within 8 days of the median interval between PN and SAE. The SAE group had significantly higher R.E.N.A.L. scores, higher N component scores, and higher L component scores (all, p <0.05). In the multivariate analysis, higher N component scores were associated with the occurrence of RAPs/AVFs (Odds ratio: 1.96, p=0.039). In the SAE group, the mean 3-day postembolization eGFR was significantly lower than the mean 3-day postoperative eGFR (p <0.01). This difference in the eGFRs was still present 1 year later.

**Conclusions:**

Renal tumors located near the renal sinus and collecting system were associated with a higher risk for RAPs/AVFs after PN. Although SAE was an effective method for controlling symptomatic RAPs/AVFs after PN, a procedure-related impairment of renal function after SAE could occur and still be present at the end of the first postoperative year.

## INTRODUCTION

Partial nephrectomy (PN) is an optimal treatment for small renal masses (1). It is associated with a lower risk of reduced renal function but also oncological outcomes and morbidities that are comparable to those associated with radical nephrectomy (2, 3). In contrast to radical nephrectomy, PN is associated with a greater risk for the development of vascular lesions and symptomatic hemorrhagic complications, namely renal artery pseudoaneurysms (RAPs) or arteriovenous fistulas (AVFs) (4, 5). RAPs and AVFs are relatively rare hemorrhagic complications. However, they are potentially life-threatening. Thus, rapid clinical evaluation and treatment are required. Until recently, some of the clinicopathologic factors in RAPs and AVFs after PN have been reported (6-8). In addition, the approach using a nephrometry scoring system to identify the correlation between tumor complexity and hemorrhagic complications has been recently introduced (9, 10). However, there is no consensus on the variables for consistent predictions of the occurrence of symptomatic hemorrhagic complications.

Selective arterial embolization (SAE) is a safe and successful method for controlling hemorrhagic complications after PN (5, 11, 12). Despite the efficacy of this procedure, few studies have addressed its effects on postsurgical renal function (8, 13). In addition, most of these studies examined renal function only within 1 to 2 weeks after SAE. Changes in renal function for at least 1 year after SAE have rarely been investigated.

Thus, the aim of the current study was to identify the clinical factors, including the R.E.N.A.L. nephrometry score, in the occurrence of RAPs and AVFs after PN. The study also sought to determine the effects of these hemorrhagic complications and SAE on post-PN renal function in the short-term and at the 1-year follow-up.

## MATERIALS AND METHODS

### Study population

The institutional review boards of three tertiary care centers in South Korea approved the retrospective study (IUBPH-18-0165, PNUH-E-2016095, KNUMC-13-1009). Between 2007 and 2016, a total of 512 patients with renal tumors underwent PN at these three institutions. Of the 512 patients, those who had multiple renal tumors resected or incomplete follow-up data were excluded. In all, 493 patients were included in the present study.

All the patients underwent either open or laparoscopic PN. Open retroperitoneal PN was performed by flank subcostal incision or eleventh rib transcostal incision. Laparoscopic PN was performed by using the standard four-port transperitoneal access. The operative technique was decided by the surgeon on the basis of tumor complexity and his experience. The renal artery alone was clamped with a Bulldog clamp, and all the procedures were performed under warm ischemia with or without intravenous mannitol administration. The renal tumor was resected by enucleoresection with a margin of approximately 0.5cm maintained around the tumor. A running medullary suture was applied after resection of tumor with absorbable polyglacin or poliglecaprone sutures. Cortical renorrhaphy by single-layer interrupted or running suturing was applied with absorbable sutures based on surgeon's experience. All the procedures were performed by five surgeons with more than 10 years of experience in PN at three tertiary care centers.

The occurrences of RAPs or AVFs were collected and analyzed. In all the patients who presented with symptoms such as gross hematuria and flank pain, biphasic contrast-enhanced computed tomography (CT) scans of the arterial and venous phases of the abdomen were performed. All symptomatic RAPs or AVFs were treated with SAE through the use of endovascular coils. The clinical success of the procedure was defined as the relief of symptoms and the lack of a need for further SAE or surgical intervention. The patients were placed into two groups: SAE and non-SAE. In the SAE group were the patients whose symptomatic vascular lesions had been treated with SAE. In the non-SAE group were those who had not experienced symptomatic RAPs or AVFs.

The clinical data on the demographic characteristics, the presence of anticoagulant therapy (either vitamin K antagonists or antiplatelet agents) for cardiovascular disease prevention and treatment, the surgical procedure, and the preoperative and follow-up visits were collected retrospectively. The patients received recommendations to discontinue any anticoagulants 5-7 days before surgery and to restart 7 days after PN.

### Preoperative R.E.N.A.L. nephrometry score calculation and glomerular filtration rate estimation based on renal function follow-up

A preoperative contrast-enhanced CT scan was used to determine the renal tumor characteristics. The R.E.N.A.L. nephrometry scoring system was retrospectively re-assessed by three urologists with experience in nephrometry scores in accordance with the published protocols (14).

The serum creatinine levels were measured at each institution. The total estimated glomerular filtration rate (eGFR) was calculated with the Modification of Diet in Renal Disease formula: GFR (mL/min/1.73 m^2^)=186 × (serum creatinine)^-1.154^ × (age)^-0.203^ × (0.742 if female) (15). The eGFR was determined 1 day before and 3 days after PN and 1 day before and 3 days after SAE. The postoperative 12-month eGFR was also determined regardless of SAE status. Any patient with an eGFR lower than 60 mL/min/1.73m^2^ was defined as having chronic kidney disease (CKD).

### Statistical Analysis

Statistical analyses were performed in SPSS, version 24 (IBM Corp., Armonk, NY, USA) and Med-Calc, version 18.9 (MedCalc Software bv, Ostend, Belgium). The variables are presented as means with standard deviations, medians with interquartile ranges (IQRs), and counts with percentages or proportions. The Mann-Whitney U test was used to compare the quantitative parameters, and Fisher's exact test was used to compare the qualitative parameters. Univariate logistic regression was used to identify the clinicopathologic factors that might have affected the occurrence of RAPs after PN. Finally, a multivariate logistic regression model, which was used in conjunction with the a standard entry method, was applied to the potential covariates. The odds ratios (ORs) and 95% confidence intervals (CIs) were determined by using the reference groups. The eGFR values at different pre-surgery and pre- and post-embolization time points were compared via a paired sample t-test. All the statistics were considered significant at a p-value of <0.05.

## RESULTS

Of the 493 patients, 33 (6.7%) experienced symptomatic RAPs or AVFs: 28 cases of RAPs and 5 of AVFs within a median of 8 days (3-113 days) between surgery and the onset of symptomatic RAPs or AVFs. None of the patients had an asymptomatic RAP or AVF that required SAE, and none of the patients with symptomatic RAPs or AVFs chose conservative treatment. Twenty-seven patients (81%) exhibited gross hematuria, and 6 (19%) complained of flank pain. The SAE group had a higher probability than the non-SAE group of having more complex tumors, as quantified by the R.E.N.A.L. nephrometry scores (intermediate complexity, 81.8% vs. 59.1%; high complexity, 9.1% vs. 2.8%; p <0.001). Similarly, an examination of the individual component scores indicated that the SAE group had more tumors closer to the collecting system (N component; p=0.019) and the renal hilum (L component; p=0.036). No significant difference was observed with regard to age, sex, tumor size, Charlson comorbidity index, pre-surgery anticoagulant therapy, or surgical approach. The characteristics of the study population are summarized in Table-1.

**Table 1:** Demographic and clinical characteristics of 493 patients.

Characteristics		SAE group (33 patients)	Non-SAE group (460 patients)	p value
**Age, years (median, IQR)**		60 (40-74)	58 (28-82)	0.376
**Gender, n (%)**				
	Male	26 (78.8)	292(63.5)	0.076
	Female	7 (21.2)	168(36.5)	
**Body mass index, kg/m^2^ (median, IQR)**		25.1 (20.2-31.2)	24.5 (16.9-33.1)	0.180
**Charlson comorbidity index, n (%)**				
	0-1	1 (3.0)	88 (19.1)	0.243
	2	11 (33.3)	117 (25.4)	
	3	10 (30.3)	98 (21.3)	
	≥4	11 (33.3)	157 (34.1)	
**Diabetes mellitus, n (%)**				
	No	28 (84.8)	388 (84.3)	0.939
	Yes	5 (15.2)	72 (15.7)	
**Hypertension, n (%)**				
	No	17 (51.5)	269 (58.6)	0.434
	Yes	16 (48.5)	191 (41.5)	
**Chronic Kidney Disease, n (%)**				
	No	30 (90.9)	444 (96.5)	0.127
	Yes	3 (9.1)	16 (3.5)	
**Anticoagulation therapy, n (%)**				
	No	26 (78.8)	398 (86.5)	0.202
	Yes	7 (21.2)	62 (13.5)	
**R.E.N.A.L score (4-6/7-9/10-12)**				
	Low complexity (4-6)	3 (9.1)	175 (38.0)	< 0.001
	Intermediate complexity (7-9)	27 (81.8)	272 (59.1)	
	High complexity (10-12)	3 (9.1)	13 (2.8)	
**Radius : maximal diameter, n (%)**				
	1 (≤ 4 cm)	27 (81.8)	405 (88.0)	0.579
	2 (between 4 and 7 cm)	6 (18.2)	45 (9.8)	
	3 (> 7 cm)	0 (0.0)	10 (2.2)	
**Exophytic/Endophytic property, n (%)**				
	1 (≥ 50 %)	11 (33.3)	215 (46.7)	0.065
	2 (< 50 %)	17 (51.5)	210 (45.7)	
	3 (entirely endophytic)	5 (15.2)	35 (7.6)	
**Nearness to collecting system or renal sinus, n (%)**				
	1 (≥ 7 mm)	2 (6.1)	99 (21.5)	0.019
	2 (between 4 and 7 mm)	3 (9.1)	58 (12.6)	
	3 (≤ 4 mm)	28 (84.8)	303 (65.9)	
**Anterior/posterior, n (%)**				
	a (anterior)	21 (63.6)	277 (60.2)	0.698
	p (posterior)	12 (36.4)	183 (39.8)	
**Location : relative to the polar line, n (%)**				
	1 (entirely superior or inferior to polar line)	11 (33.3)	255 (55.4)	0.036
	2 (crosses polar line)	10 (30.3)	85 (18.5)	
	3 (>50% of mass is across polar line Or mass crosses a axial midline Or mass is between polar lines)	12 (36.4)	120 (26.1)	
**Tumor size, cm (median, IQR)**		2.7 (0.8-5.4)	2.5 (0.8-5.2)	0.643
**Malignant tumor, n (%)**				
	No	4 (12.1)	61 (13.3)	1.000
	Yes	29 (87.9)	399 (86.7)	
**Positive surgical margin, n (%)**				
	No	31 (93.9)	437 (95.0)	0.680
	Yes	2 (6.1)	23 (5.0)	
**Surgical approach, n (%)**				
	Open	18 (54.5)	256 (55.7)	0.902
	Laparoscopic	15 (45.5)	204 (44.3)	
**Medullary suture, n (%)**				
	No	1 (3.0)	11 (2.4)	0.569
	Yes	32 (97.0)	449 (97.6)	
**Estimated blood loss, mL (median, IQR)**		420 (80-1200)	400 (50-1237)	0.097
**Warm ischemic time, min (median, IQR)**		21 (12-42)	20 (5-44)	0.225

**SAE** = selective arterial embolization; **IQR** = interquartile range

In the univariate analysis, the higher N component score (OR: 2.06; CI, 1.09-3.88; p=0.026) and the higher L component score (OR: 1.52; CI, 1.02-2.26; p=0.039) were risk factors for RAPs or AVFs (Table-2). The Charlson comorbidity index, preoperative anticoagulant therapy, and surgical approach were not predictive of the occurrence of RAPs or AVFs. In the multivariate analysis, the higher N component score (OR: 1.96; CI, 1.04-3.71; p=0.039) was associated with a significantly increased risk of RAPs or AVFs.

**Table 2:** Univariate and multivariate logistic regression analysis of factors influencing the occurrence of RAP or AVF after partial nephrectomy.

		Univariate			Multivariate		
		OR	95% CI	p value	OR	95% CI	p value
Age		1.02	(0.98-1.05)	0.38		
Gender (male/female)		0.47	(0.19-1.10)	0.08		
Body mass index		1.07	(0.97-1.18)	0.18		
Charlson comorbidity index (every 1unit increase)		1.13	(0.93-1.38)	0.23		
Diabetes mellitus (no/yes)		0.96	(0.36-2.58)	0.94		
Hypertension (no/yes)		1.33	(0.65-2.69)	0.44		
Chronic Kidney Disease (no/yes)		2.78	(0.77-10.05)	0.12		
Anticoagulation therapy (no/yes)		1.73	(0.72-4.15)	0.22		
**R.E.N.A.L score**						
	R (every 1unit increase)	1.25	(0.57-2.73)	0.58		
	E (every 1unit increase)	1.65	(0.96-2.82)	0.07		
	N (every 1unit increase)	2.06	(1.09-3.88)	0.026	1.96	(1.04-3.71)	0.039
	A (Anterior/Posterior)	0.87	(0.42-1.80)	0.69		
	L (every 1unit increase)	1.52	(1.02-2.26)	0.039	1.44	(0.96-2.16)	0.075
Tumor size		1.06	(0.82-1.37)	0.64		
Malignant tumor (no/yes)		1.11	(0.38-3.26)	0.85		
Positive surgical margin (no/yes)		1.23	(0.28-5.44)	0.79		
Surgical approach (Open/Laparoscopic)		1.05	(0.51-2.13)	0.90		
Medullary suture (no/yes)		0.78	(0.10-6.26)	0.82		
Estimated blood loss		1.00	(1.00-1.00)	0.09		
Warm ischemic time		1.02	(0.99-1.06)	0.22		
eGFR (Pre-operative)		0.99	(0.98-1.01)	0.58		

**eGFR** = estimated glomerular fltration rate

**Figure 1:**
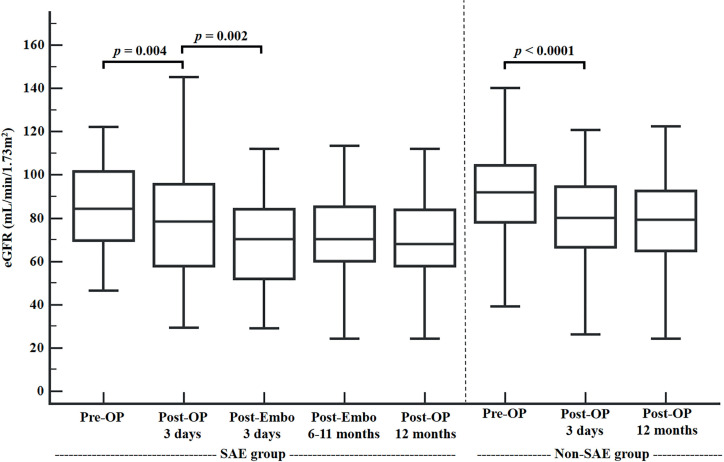
Mean eGFR values and paired comparison at different time points before surgery, before and after embolization

All RAPs and AVFs were successfully treated in a single SAE session. Table-3 and Figure-1 show the mean eGFR values and a paired comparison of the eGFR values of the entire cohort at different time points before surgery, as well as before and after SAE. The mean 3-day postoperative eGFRs for the SAE and non-SAE groups were 77.8±25.3mL/min/1.73m^2^ and 79.4±20.2mL/min/1.73m^2^, which were lower than the mean preoperative eGFRs (85.7±26.7mL/min/1.73m^2^ and 91.6±20.7mL/min/1.73m2, respectively). This reflected a loss of functional renal parenchyma during PN. In the SAE group, the mean postembolization eGFR values at 3 days, 6-11 months, and 12 months postoperative were significantly lower than the mean 3-day postoperative eGFR values (70.5±26.7, 70.7±20.9, and 69.4±19.9mL/min/1.73m^2^ vs. 77.8±25.3mL/min/1.73m2, all, p <0.01). This difference was still present at the 1-year postoperative follow-up. However, the mean 3-day postoperative eGFR values in the non-SAE group were still the same at the 1-year postoperative follow-up.

**Table 3:** Mean eGFR values and paired comparison at different time points before surgery, before and after embolization.

		SAE group (33 patients)			Non-SAE group (460 patients)		
Time point		Mean eGFR (mL/min/1.73m^2^)	95% CI	ST. Dev.	Mean eGFR (mL/min/1.73m^2^)	95% CI	ST. Dev.
	Pre-operative	85.7	76.3-95.2	26.7	91.6	88.7-94.4	20.7
	Post-operative 3 days	77.8	68.8-86.8	25.3	79.4	76.6-82.1	20.2
	Post-embolization 3 days	70.5	61.1-79.9	26.7	-	-	-
	Post-embolization 6-11 months	70.7	63.2-78.1	20.9	-	-	-
	Post-operative 12 months	69.4	62.4-76.5	19.9	79.2	76.5-81.8	19.1
Paired comparison		t value	df	Sig (two-tailed)	t value	df	Sig (two-tailed)
	Pre-operative vs. Postoperative 3 days	-3.05	32	0.004	-14.56	459	< 0.001
	Post-operative 3 days vs. Post-embolization 3 days	-3.35	32	0.002	-	-	-
	Post-operative 3 days vs. Post-embolization 6-11 months	-3.85	32	< 0.001	-	-	-
	Post-operative 3 days vs. Post-operative 12 months	-2.91	32	0.006	-0.15	459	0.88

## DISCUSSION

RAPs and AVFs are rare postoperative complications of PN. The reported incidence after PN ranges from 0.43% to 2.6% for both open and laparoscopic PNs (10). In the current study, 6.7% patients were identified as being affected by these symptomatic hemorrhagic complications after nephron sparing surgery (NSS). The incidence was higher than that in previous studies. The reason might have been the proactive inspection and intervention upon the initial presentation of symptoms.

The precise etiology of these hemorrhagic complications is unknown. However, RAPs are thought to arise from renal artery branch transection or puncturing during tumor resection and suture ligation of the resection bed (4, 16). The damaged renal artery branch is initially covered by vascular adventitia, renal parenchyma, or hematoma. However, a few days later, high pressure arterial blood eventually extravasates into the extravascular space and adjacent collecting system, causing perirenal hematoma or hematuria. Similarly, an AVF may form when both an artery and a nearby vein are injured, resulting in blood crossing from a higher pressure system directly into the adjacent vein (4, 11).

A nephrometry scoring system was initially developed to assess the overall complexity and resectability of renal tumors. Of the various kinds of nephrometry scores, the R.E.N.A.L. nephrometry score is the most widely used for preoperatively defining renal masses (14, 17). Several previous studies have proposed that there is a correlation between nephrometry scores and the incidence of RAPs. However, the predictive value of the R.E.N.A.L. nephrometry scoring system for the occurrence of hemorrhagic complications after PN is controversial. A recent study by Jung et al. failed to demonstrate an association between the R.E.N.A.L. nephrometry score and bleeding risk (7). The results of a univariate analysis in a subsequent study by Omae et al. found the N component in the R.E.N.A.L. nephrometry score to be a statistically significant predictor of RAPs. However, the results of a multivariate analysis showed significance for only renal sinus exposure (9). Although Gupta et al. did not directly evaluate the risk of RAP formation, their multivariate analysis indicated that a higher R.E.N.A.L. score is associated with an increasing likelihood of multiple RAPs (10). Similarly, our results also confirmed that patients with renal tumors that are closer to the renal sinus or collecting system have a higher incidence and probability of experiencing RAPs or AVFs. The main clinical relevance of these results is the need for careful suturing of the renal bed, such as the early unclamping of the renal artery prior to renorrhaphy during PN (18). In addition, close observation is necessary during postoperative care in the cases in which more complex and centralized tumors develop.

The statistical model in the present study indicated that the other factors, including preoperative anticoagulant treatment, comorbidities, and pathologic characteristics, were not significant. Although robot-assisted PN was not included, no difference in the RAP or AVF occurrence rate was observed with the open and laparoscopic approaches. However, a systematic review reported that after minimally invasive PN, the incidence of RAPs was higher than that after open approaches (5). This discrepancy could be attributed to several factors, including the effects of the surgeon's experience in minimally invasive PN and the various suturing techniques during PN (19, 20). Therefore, a further well-designed systematic review is warranted for selecting an appropriate surgical approach that lowers the incidence of complications.

SAE for controlling renal biopsy-related AVFs was first reported in 1973 (21). Since then, it has become the first-line therapy for iatrogenic vascular lesions after urologic procedures, such as PN and percutaneous nephrolithotomy. Several studies have demonstrated the safety and efficacy of SAE for controlling hemorrhagic complications after PN (4, 10, 22). In the present study, 33 patients who had symptomatic RAPs underwent SAE with 100% technical success. The major advantage of this study is the observation of the effects of SAE on postoperative renal function in serial. Despite several previous reports of the efficacy of SAE in RAPs, studies on post-SAE serial renal function have rarely been reported. In the present study, the mean postembolization eGFR values at different time points after embolization were significantly different from the mean postoperative eGFR for those who did not experience RAP. This finding suggests that SAE has adverse effects on renal function despite its usefulness in controlling postoperative hemorrhagic complications.

Two possible mechanisms in SAE-induced reduction of postoperative renal function have been proposed. First, the loss of renal parenchyma after SAE is the primary reason for decreased renal function after the procedure. Theoretically, SAE is safe for preserving renal function because of the selective treatment of damaged and bleeding vessels (23). However, the increased devascularized renal parenchyma after a procedure cannot be avoided because all the arterioles, including the segmental arteries, inter-lobular arteries, and arcuate arteries in the kidney, are anatomic end arteries. Indeed, a recent CT-based renal parenchymal volumetric analysis showed that 25% of the parenchymal volume was reduced at a median 100 days after embolization (22). Second, the contrast media during the procedure could affect post-procedure renal function. Gupta et al. proposed a possible mechanism in the relationship between contrast media and decreased renal function (10). During the immediate postoperative period, the kidney is recovering from ischemia; thus, the administration of nephrotoxic contrast media during this vulnerable period could worsen renal parenchymal damage.

The present study has several limitations. First, it used a retrospective design with a small patient cohort. However, the multi-institutional research and surgical cases performed on various surgeons were used to generalize the frequency of RAPs or AVFs in clinical settings. Second, a nuclear renal scan was not used to detect renal function changes after PN and SAE. In addition, because of the artifacts associated with the coiling material that is used in SAE on CT scans, CT-based volumetric analysis could not be performed to detect SAE-related parenchymal devascularization. Finally, open and laparoscopic PNs were addressed in the analysis; however, robot-assisted PN was not included. Because robot-assisted NSS is performed more frequently, further analyses on robot-assisted PN are necessary.

## CONCLUSIONS

Renal tumors located near the renal sinus and collecting system have been associated with a higher risk for RAPs or AVFs. Although SAE was an effective method for controlling symptomatic hemorrhagic complications after PN, a procedure-related impairment of renal function after SAE could occur and still be present at the postoperative 1-year follow-up. Therefore, the preliminary explanation regarding the procedure related renal function impairment before SAE is essential in real clinical circumstance.

## References

[1] 1. Klatte T, Ficarra V, Gratzke C, Kaouk J, Kutikov A, Macchi V, et al. A Literature Review of Renal Surgical Anatomy and Surgical Strategies for Partial Nephrectomy. Eur Urol. 2015;68:980-92. 10.1016/j.eururo.2015.04.010 PMC4994971 25911061

[2] 2. Zini L, Perrotte P, Capitanio U, Jeldres C, Shariat SF, Antebi E, et al. Radical versus partial nephrectomy: effect on overall and noncancer mortality. Cancer. 2009;115:1465-71. 10.1002/cncr.24035 19195042

[3] 3. Tobert CM, Riedinger CB, Lane BR. Do we know (or just believe) that partial nephrectomy leads to better survival than radical nephrectomy for renal cancer? World J Urol. 2014;32:573-9. 10.1007/s00345-014-1275-8 24671608

[4] 4. Hyams ES, Pierorazio P, Proteek O, Sukumar S, Wagner AA, Mechaber JL, et al. Iatrogenic vascular lesions after minimally invasive partial nephrectomy: a multi-institutional study of clinical and renal functional outcomes. Urology. 2011;78:820-6. 10.1016/j.urology.2011.04.063 21813164

[5] 5. Jain S, Nyirenda T, Yates J, Munver R. Incidence of renal artery pseudoaneurysm following open and minimally invasive partial nephrectomy: a systematic review and comparative analysis. J Urol. 2013;189:1643-8. 10.1016/j.juro.2012.11.170 23219544

[6] 6. Fardoun T, Chaste D, Oger E, Mathieu R, Peyronnet B, Rioux-Leclercq N, et al. Predictive factors of hemorrhagic complications after partial nephrectomy. Eur J Surg Oncol. 2014;40:85-9. 10.1016/j.ejso.2013.11.006 24268762

[7] 7. Jung S, Min GE, Chung BI, Jeon SH. Risk factors for postoperative hemorrhage after partial nephrectomy. Korean J Urol. 2014;55:17-22. 10.4111/kju.2014.55.1.17 PMC3897624 24466392

[8] 8. Chavali JSS, Bertolo R, Kara O, Garisto J, Mouracade P, Nelson RJ, et al. Renal Arterial Pseudoaneurysm After Partial Nephrectomy: Literature Review and Single-Center Analysis of Predictive Factors and Renal Functional Outcomes. J Laparoendosc Adv Surg Tech A. 2019;29:45-50. 10.1089/lap.2018.0364 30300074

[9] 9. Omae K, Kondo T, Takagi T, Morita S, Hashimoto Y, Kobayashi H, et al. Renal sinus exposure as an independent factor predicting asymptomatic unruptured pseudoaneurysm formation detected in the early postoperative period after minimally invasive partial nephrectomy. Int J Urol. 2015;22:356-61. 10.1111/iju.12696 25581594

[10] 10. Gupta N, Patel A, Ensor J, Ahrar K, Ahrar J, Tam A, et al. Multiple Renal Artery Pseudoaneurysms in Patients Undergoing Renal Artery Embolization Following Partial Nephrectomy: Correlation with RENAL Nephrometry Scores. Cardiovasc Intervent Radiol. 2017;40:202-9. 10.1007/s00270-016-1473-4 27681271

[11] 11. Ghoneim TP, Thornton RH, Solomon SB, Adamy A, Favaretto RL, Russo P. Selective arterial embolization for pseudoaneurysms and arteriovenous fistula of renal artery branches following partial nephrectomy. J Urol. 2011;185:2061-5. 10.1016/j.juro.2011.02.049 21496835

[12] 12. Gahan JC, Gaitonde M, Wadskier L, Cadeddu JA, Trimmer C. Renal function outcomes following selective angioembolization for iatrogenic vascular lesions after partial nephrectomy. J Endourol. 2013;27:1516-9. 10.1089/end.2013.0201 24199730

[13] 13. Guo H, Wang C, Yang M, Tong X, Wang J, Guan H, et la. Management of iatrogenic renal arteriovenous fistula and renal arterial pseudoaneurysm by transarterial embolization: A single center analysis and outcomes. Medicine (Baltimore). 2017;96:e8187. 10.1097/MD.0000000000008187 PMC5738006 28984770

[14] 14. Kutikov A, Uzzo RG. The R.E.N.A.L. nephrometry score: a comprehensive standardized system for quantitating renal tumor size, location and depth. J Urol. 2009;182:844-53. 10.1016/j.juro.2009.05.035 19616235

[15] 15. Kidney Disease: Improving Global Outcomes (KDIGO) CKD Work Group.: Definition and classification of CKD. Kidney Int Suppl. 2013; 3:19-62.

[16] 16. Takagi T, Kondo T, Tajima T, Campbell SC, Tanabe K. Enhanced computed tomography after partial nephrectomy in early postoperative period to detect asymptomatic renal artery pseudoaneurysm. Int J Urol. 2014;21:880-5. 10.1111/iju.12462 24712736

[17] 17. Sterzik A, Solyanik O, Eichelberg C, Jost M, Graser A, Lausenmeyer EM, et al. Improved prediction of nephronsparing surgery versus radical nephrectomy by the optimized R.E.N.A.L. Score in patients undergoing surgery for renal masses. Minerva Urol Nefrol. 2019;71:249-57. 10.23736/S0393-2249.18.03134-X 30256079

[18] 18. Delto JC, Chang P, Hyde S, McAnally K, Crociani C, Wagner AA. Reducing Pseudoaneurysm and Urine Leak After Robotic Partial Nephrectomy: Results Using the Early Unclamping Technique. Urology. 2019;132:130-135. 10.1016/j.urology.2019.05.042 31254571

[19] 19. Bertolo R, Campi R, Klatte T, Kriegmair MC, Mir MC, Ouzaid I, et al. Suture techniques during laparoscopic and robot-assisted partial nephrectomy: a systematic review and quantitative synthesis of peri-operative outcomes. BJU Int. 2019;123:923-46. 10.1111/bju.14537 30216617

[20] 20. Larcher A, Muttin F, Peyronnet B, De Naeyer G, Khene ZE, Dell'Oglio P, et al. The Learning Curve for Robot-assisted Partial Nephrectomy: Impact of Surgical Experience on Perioperative Outcomes. Eur Urol. 2019;75:253-6. 10.1016/j.eururo.2018.08.042 30243798

[21] 21. Bookstein JJ, Goldstein HM. Successful management of postbiopsy arteriovenous fistula with selective arterial embolization. Radiology. 1973;109:535-6. 10.1148/109.3.535 4772158

[22] 22. Strobl FF, D'Anastasi M, Hinzpeter R, Franke PS, Trumm CG, Waggershauser T, et al. Renal Pseudoaneurysms and Arteriovenous Fistulas as a Complication of Nephron-Sparing Partial Nephrectomy: Technical and Functional Outcomes of Patients Treated With Selective Microcoil Embolization During a Ten-Year Period. Rofo. 2016;188:188-94. 10.1055/s-0041-110136 26756934

[23] 23. Poulakis V, Ferakis N, Becht E, Deliveliotis C, Duex M. Treatment of renal-vascular injury by transcatheter embolization: immediate and long-term effects on renal function. J Endourol. 2006;20:405-9. 10.1089/end.2006.20.405 16808653

